# Contribution of estradiol levels and hormonal contraceptives to sex differences within the fear network during fear conditioning and extinction

**DOI:** 10.1186/s12888-015-0673-9

**Published:** 2015-11-18

**Authors:** Moon Jung Hwang, Rachel G. Zsido, Huijin Song, Edward F. Pace-Schott, Karen Klahr Miller, Kelimer Lebron-Milad, Marie-France Marin, Mohammed R. Milad

**Affiliations:** Department of Psychiatry, Massachusetts General Hospital & Harvard Medical School, CNY 149 13th Street Room 2508, Charlestown, Boston, MA 02129 USA; Department of Neuroendocrine Unit, Department of Medicine, Massachusetts General Hospital and Harvard Medical School, Boston, MA 02114 USA

**Keywords:** Gonadal hormones, Estrogens, Estradiol, Sex differences, Fear conditioning, Extinction memory, Men, Women, Conditioned response, Unconditioned response, fMRI

## Abstract

**Background:**

Findings about sex differences in the field of fear conditioning and fear extinction have been mixed. At the psychophysiological level, sex differences emerge only when taking estradiol levels of women into consideration. This suggests that this hormone may also influence sex differences with regards to activations of brain regions involved in fear conditioning and its extinction. Importantly, the neurobiological correlates associated with the use of hormonal oral contraceptives in women have not been fully contrasted against men and against naturally cycling women with different levels of estradiol. In this study, we begin to fill these scientific gaps.

**Methods:**

We recruited 37 healthy men and 48 healthy women. Of these women, 16 were using oral contraceptives (OC) and 32 were naturally cycling. For these naturally cycling women, a median split was performed on their serum estradiol levels to create a high estradiol (HE) group (*n* = 16) and a low estradiol (LE) group (*n* = 16). All participants underwent a 2-day fear conditioning and extinction paradigm in a 3 T MR scanner. Using the 4 groups (men, HE women, LE women, and OC users) and controlling for age and coil type, one-way ANCOVAs were performed to look at significant activations within the nodes of the fear circuit. Using post-hoc analyses, beta-weights were extracted in brain regions showing significant effects in order to unveil the differences based on hormonal status (men, HE, LE, OC).

**Results:**

Significant main effect of hormonal status group was found across the different phases of the experiment and in different sub-regions of the insular and cingulate cortices, amygdala, hippocampus, and hypothalamus. During conditioning, extinction and recall, most of the observed differences suggested higher activations among HE women relative to men. During the unconditioned response, however, a different pattern was observed with men showing significantly higher brain activations.

**Conclusions:**

Our data further support the important contribution of estradiol levels in the activation of brain regions underlying fear learning and extinction. The results highlight the need to document gonadal hormonal levels, menstrual cycle phase as well as oral contraceptive use in women in order to avoid overlooking sex differences when investigating the neurobiology of emotional regulation.

## Background

The neural correlates of the acquisition and extinction of conditioned fear are relatively well described in humans. The amygdala, hippocampus, insular cortex, and dorsal anterior cingulate cortex (dACC) are regions known to show significant activations in response to the presentation of conditioned fear cues [1–6]. The hypothalamus, amygdala, hippocampus, orbitofrontal cortex, and medial prefrontal cortex are also known to show significant activations during unconditioned fear cues [7]. Subsets of these brain areas along with the ventromedial prefrontal cortex (vmPFC) show robust activations during extinction learning and extinction memory recall [5, 8, 9]. Resting metabolism and functional coupling within this fear extinction network are associated with the expression of conditioned responses in healthy individuals [10, 11].

Importantly, the pathophysiology of numerous anxiety and fear-based disorders has been associated with dysfunctional activations of this network in the context of fear extinction [12–15]. It is also well established that the prevalence of anxiety and fear-based disorders is higher in women [16–19] and that the brain regions noted above are sexually dimorphic [20–22]. Reports related to sex differences during fear learning and extinction are often inconsistent, highlighting the need to examine biological factors that may contribute to this inconsistency [22–27]. These studies call for a better understanding of how sex differences may emerge during fear conditioning and extinction, as well as how sex hormones may mediate some of these differences [23–30]. Estrogens are a class of gonadal hormones that include estrone, estriol and estradiol. Estradiol is the predominant and most potent circulating estrogen during the reproductive years in non-pregnant women.

A recent preliminary study using functional magnetic resonance imaging (fMRI) reported sex differences in the activation of the amygdala, dACC, and vmPFC during fear conditioning, extinction, and recall [23]. Despite the lack of sex differences in skin conductance responses during fear conditioning and extinction memory recall, women exhibited significantly greater activation within the amygdala and dACC during fear conditioning while men showed significantly greater activation of the vmPFC during extinction recall [23]. When taking into account the hormonal milieu, it has been shown that elevated estradiol levels are associated with increased vmPFC, amygdala, and hippocampal activation during extinction recall in women [29]. In addition, exogenous administration of estradiol to women [28, 30] and to female rats [27, 28, 31] has been shown to increase the consolidation of extinction memory**.** What remains to be tested is how variance in estradiol levels in women could contribute to the presence, or absence, of sex differences during fear regulation.

The studies mentioned above examined the role of estradiol in fear extinction in naturally cycling subjects. Relatively few studies have examined the effects of hormonal contraceptive use on fear extinction. Combined oral contraceptives contain ethinyl estradiol and progestin, which inhibit ovulation by decreasing ovarian production of estrogens and progesterone. This leads to a sustained reduction in the level of circulating estrogens comparable to levels found in naturally cycling women during the early follicular (i.e. low gonadal hormones) phase of the menstrual cycle [32]. As mentioned above, oral contraceptives contain ethinyl estradiol, a synthetic estrogen that binds to estrogen receptors at levels high enough to prevent ovulation through negative feedback at the hypothalamus and pituitary gland [32]. It is therefore unclear if oral contraceptive use exacerbates or diminishes sex differences in the reactivity of the fear extinction network.

In the present study, we conducted a comprehensive analysis of fMRI data from a cohort of women who underwent extinction learning either during high or low estradiol states or while using oral contraceptives. We also included a cohort of men. All subjects underwent a validated 2-day fear conditioning and extinction paradigm [23, 28, 29]. Some of the psychophysiological and fMRI data pertaining to fear extinction have been previously published [28–30]. In the present study, we focused our analysis on brain activation to specifically explore the presence or absence of sex/hormonal status differences in response to unconditioned and conditioned stimuli during fear and extinction learning and extinction recall. We primarily studied the activation of the insular cortex, cingulate cortex, amygdala, hippocampus, vmPFC, and hypothalamus. We analyzed the brain activation to the conditioned stimuli during the fear acquisition, extinction learning, and extinction recall phases as well as the unconditioned responses during the conditioning phase. Moreover, comparisons of the psychophysiological data across the different groups of women and men have been previously reported [23, 28, 30] and as such will not be included in the present study.

## Methods

### Participants

Data from a total of 85 healthy right-handed individuals were selected from a large pool of subjects that had participated in studies within our lab over the past few years. All participants were without neurologic, endocrine, or other medical conditions and were evaluated and screened for Axis I mental disorders via the Structured Clinical Interview for DSM-IV [33, 34]. No participants were using psychoactive or other potentially confounding drugs or medications. There were a total of 37 men and 48 women. The selection of all women out of our large database was based on our knowledge of their levels of estradiol and/or our documented data on their oral contraceptive use. Thus, women from whom we did not have these data were excluded from the analysis. The composition of the women included 32 naturally cycling women and 16 women using monophasic oral contraceptives (OC) for at least 3 months. The naturally cycling group was divided into 2 groups based on serum estradiol levels measured at the day of fear extinction learning just prior to the fMRI scan. A median split of 108 pg/mL was used to separate the 32 naturally cycling women into 16 high estradiol women (HE) and 16 low estradiol (LE) women. The average estradiol level of naturally cycling women was 138 pg/mL (SD = 116.5) and the average estradiol level per group was 222.6 pg/mL (SD = 109.0) and 53.7 pg/mL (SD = 30.9), respectively. Mean age and years of education as a function of group are summarized in Table 1. One-way MANOVAs performed on age and years of education with Group (men, HE, LE, OC) as the between-subject factor confirmed a significant effect of group for age, F(3,80) = 7.52, *p* < 0.001. Age was therefore added as a covariate for all analyses. All procedures were approved by Partners Healthcare Institutional Review Board and written informed consent was obtained from all participants in accordance with requirements of the Partners Healthcare Human Research Committee. As mentioned above, portions of these data from the cohorts included in our analyses were used for prior publications by our group [28, 30].

**Table 1:** Demographic information for the study population

	*N*		Age (SD)	Education (SD)	Estradiol (SD)
Men		37	29.8 (8.8)	16.4 (2.2)	
Women		48	23.3 (2.4)	15.9 (1.1)	
Women	HE	16	23.0 (2.7)	15.8 (1.0)	222.6 (109.0)
	LE	16	23.4 (2.6)	15.9 (1.2)	53.7 (30.9)
	OC	16	23.6 (1.8)	15.9 (1.2)	
All		85	26.2 (6.8)	16.1 (1.7)	

Sample size (*n*) and means for age, years of education, and estradiol levels as a function of group are indicated. SD = standard deviations. Age showed a significant effect of Group and was therefore added as a covariate in all analyses

### Fear conditioning, extinction, and recall procedures

All participants underwent our validated 2-day fear conditioning and extinction procedure that has been previously described [23, 28, 29]. Day 1 consisted of the habituation, fear conditioning, and extinction phases. Approximately 24 h later, subjects underwent the extinction recall test. The conditioned stimuli (CSs) were pictures of lamps (i.e. blue, red, and yellow lights) that appeared within 1 of 2 rooms (i.e. an office or library) that served as the fear conditioning and extinction learning contexts. The unconditioned stimulus (US) was a mild electric shock delivered to the second and third finger of the subjects’ right hands. The subjects selected their own level of shock that they found to be “highly annoying but not painful.” At the initiation of the experiment, subjects underwent 6 trials of habituation (CS alone) immediately followed by a total of 32 trials during the conditioning phase. In the fear-conditioning phase, 2 of the CSs were each presented 8 times with 62.5 % partial reinforcement (CS + s, five shocks for each), while the third CS was never followed by a shock (CS-). The CS- was presented 16 times intermingled with the 2 CS + s. In each trial, the context images were presented for a total of 9 s: 3 s with the lights off followed by 6 s with the light on. The mean inter-trial interval was 15 s, with a range of 12 s to 18 s. The shock delivery occurred immediately at the offset of the CS+ and lasted for 0.5 s. The conditioning phase was immediately followed by extinction learning during which one of the CS + s was presented without the US, in a different context than the one used during conditioning (i.e. the extinction context). During this phase, the CS+ that was extinguished (CSE) was presented 16 times intermingled with 16 presentations of the CS-. The following day, extinction memory recall was tested by presenting all 3 CSs (CSE, CS-, and the unextinguished CS+ (CSU)) without any US in the extinction-learning context.

### Image acquisition

A 3.0 Tesla Siemens MAGNETOM Trio, whole body MRI system imaging device (Siemens Medical Systems, Iselin, New Jersey) was used to acquire whole brain images with conventional 12-channel or 32-channel head coils. Subjects were instructed to lie as still as possible and head movement was restricted with foam cushions. After an automated scout image was obtained, a high-resolution T1-weighted anatomic image was acquired using three-dimensional magnetization-prepared rapid acquisition multi gradient echo (MEMPRAGE) for structural reference to facilitate spatial normalization. fMRI blood oxygenation level dependent (BOLD) images were acquired with an interleaved T2*-weighted EPI sequence (TR = 3000 ms, TE = 30 ms, Flip angle = 90°), oblique axial along with the anterior-posterior commissure line to cover the whole brain using the 12-channel coil. For the 32-channel coil, images were acquired with the following parameters (TR = 2560 ms, TE = 30 ms, Flip angle = 90°) collected to cover the whole brain. Here is the distribution of subjects as a function of coil type: 26 men with 12-channel coil and 11 men with 32-channel coil; 16 LE women with 12-channel coil; 16 HE women with 12-channel coil; 9 OC users with 12-channel coil and 7 OC users with 32-channel coil. Given the unequal distribution of subjects as a function of head coil, this factor was used as a covariate in the fMRI between-group analyses.

### Functional MRI data analysis

Image processing and statistical analyses were performed using Matlab v2012b (The Mathworks Inc, Natick, Massachusetts, USA) and Statistical Parametric Mapping (SPM8; Wellcome Trust Centre for Neuroimaging, www.fil.ion.ucl.ac.uk) for all MRI data. Structural images were segmented and spatially normalized to the Montreal Neurological Institute (MNI) T1 template. Functional images were corrected for slice timing, realigned, co-registered with the structural image, normalized into MNI space using parameters obtained from the structural normalization process, and finally smoothed with an 8 mm full width half-maximum Gaussian kernel to increase the signal-to-noise ratio and account for anatomical variations between subjects. High-pass temporal filtering with a cutoff of 128 s was applied to remove low frequency signal drift. Serial correlations in the fMRI time series due to aliased biorhythms were accounted for using an autoregressive AR(1) model. Artifact detection toolbox (ART, http://gablab.mit.edu) was applied to detect a spike and spiking motion in the functional temporal data. The motion artifact data detected by ART were used in the first-level analysis as regressors with movement parameters (x, y, z, roll, pitch, and yaw) from the realignment process. Data from subjects that exhibited movement greater than 3 mm or 3° were excluded from all subsequent analyses. For the remainder of the subjects, motion regressors were generated using the ART-tool with condition of movement 1 mm and rotation 0.087 radian in SPM and applied to all first-level analyses. These criteria resulted in the exclusion of 13 subjects (4 men, 1 HE, 4 LE, and 4 OC) for conditioning, 17 subjects (6 men, 2 HE, 5 LE, and 4 OC) for extinction, and 16 subjects (9 men, 2 HE, 3 LE, and 2 OC) for the recall phase of the fMRI analysis.

### First-level model

After preprocessing, each subject’s functional time series was modeled for each experimental phase using a general linear model specifying the condition onsets. For the conditioning phase, these conditions included all CS+ trials, CS- trials, shocks (i.e., the 0.5 s shock delivered at the offset of 10 of the 16 CS+ trials), and CS- offsets (the offset of the 16 CS- trials, when the shock was never expected and was never delivered). Extinction phase onsets were modeled for the late CSE and CS-, i.e., the last 8 trials of the 16 CSE trials and the last 8 trials of the 16 CS- trials. Extinction recall phase onsets were modeled for the first four trials of CSE and CSU. Movement parameters from the realignment step and ART regressor (described above) were included in the model to remove residual motion-related noise. Activated voxels in each experimental phase were identified using a statistical model containing event-related design functions representing each of the experimental conditions with the SPM canonical hemodynamic response function.

Based on prior literature, we focused our attention on 4 different contrasts of interest: 1) the unconditioned response during the fear conditioning phase (UCR: Shock vs. CS- offset) in order to examine activations associated with the sensation of the shock; 2) the conditioned response during the fear conditioning phase (Conditioning: CS+ vs. CS-) in order to examine fear conditioning-induced activations; 3) the late extinction phase (last 8 trials of CSE vs. last 8 trials of CS-) in order to examine fear extinction learning–induced activation towards the end of extinction learning; and 4) the early extinction recall phase (first 4 trials of CSE vs. first 4 trials of CSU) in order to examine extinction memory-induced brain activation during the early phase of recall.

### Second-level model and ROI selection

The first-level contrasts listed above were obtained for each subject, and were modeled at the second-level using a general linear model and random effect analysis. Our main objective was to examine how hormonal status affects the activation of the fear network. To this end, we first conducted a main-effect analysis with a one-way ANCOVA across all groups (men, HE, LE and OC). Because age and coil type differ between groups, they were used as covariates to control any potential confounding effects on activations in the fear extinction network. Based on prior literature, we were specifically interested in the insular cortex, cingulate cortex (both middle [MCC] and anterior [ACC]), amygdala, hippocampus, and hypothalamus. A *p* < 0.05 threshold with a minimum of 20 contiguous voxels criterion was used to detect any significant results within these regions. Clusters detected within these regions that survived small volume family-wise error (FWE) correction (*p* < 0.05) were considered significant. For those clusters that survived the correction, we extracted beta weights using REX (region of interest extraction) toolbox (http://web.mit.edu/swg/software.htm) (representing BOLD effect) for each region of interest (ROI) found to exceed the threshold noted above. Bonferroni post-hoc t-tests were conducted on the extracted beta values to assess significant group differences between men, HE, LE and OC women. SPSS (v21) software was used for post-hoc group comparisons of the extracted beta values.

### Correlations between estradiol levels and brain activations

For the sake of completeness, we performed a regression analysis for each of the above-mentioned contrast. Importantly, serum estradiol levels were only documented in naturally-cycling women and we therefore combined those two subgroups for that specific analysis and used serum estradiol levels as a regressor. Age was added as a covariate. Note that coil type was not used as a covariate here given that all naturally cycling women were scanned with 12-channel coil. An initial threshold of 20 contiguous voxels and *p* < 0.05 was used to detect positive or negative correlations in the brain regions involved in fear acquisition and extinction. Clusters detected with this initial threshold that survived small volume FWE correction (*p* < 0.05) were considered significant.

## Results

Overall, the insular and cingulate cortices showed significant differences in terms of activation as a function of Group across the different phases of the experiment (conditioning, extinction, and extinction recall). In addition, the amygdala and the hypothalamus showed significant differences among the groups only during the conditioned response of the fear-conditioning phase. During the unconditioned response of the fear-conditioning phase, significant effects were revealed in the hippocampus and the insular cortex. Details of these results are described below. Note that p values reported are FWE corrected. The cluster size reported is the number of voxels that survived the FWE correction, thus explaining why some cluster sizes are smaller than 20.

### Sex differences in the insular cortex

**Fig. 1:**
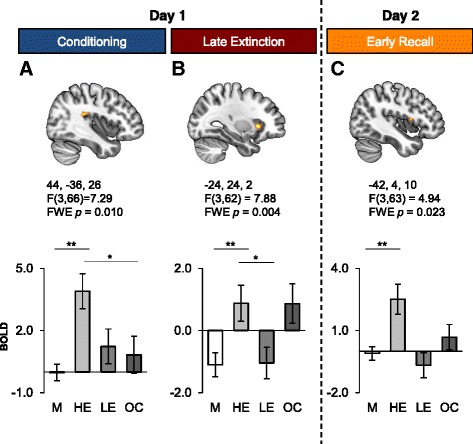
Insular cortex activations as a function of hormonal status grouping and phases of the fear conditioning and extinction paradigm. Significant functional activations of the insular cortex are displayed as a function of the different phases: fear conditioning (**a**), late extinction learning (**b**), and early extinction recall (**c**). The Montreal Neurological Institute (MNI) coordinates are listed below each functional activation, along with the corresponding statistics (note that the reported p values are FWE corrected). The lower panel indicates the BOLD signal extracted from the corresponding cluster as a function of the different groups during conditioning (**a**), late extinction (**b**), and early recall (**c**). FWE: family-wise error, M: men, HE: high estradiol women, LE: low estradiol women, OC: oral contraceptive users. Significant differences are noted (* *p*  0.05 ** *p* < 0.01)

Across the 3 phases of the study (conditioning, extinction, and extinction recall), the one-way ANCOVAs revealed a significant main effect of Group in different sub-regions of the insular cortex (IC) (Fig. 1). During fear conditioning, a main effect of Group was noted in the posterior IC (left: −40, −40, 16; cluster size = 23; F(3,66) = 6.03, *FWE p* = 0.029 (data not shown), right: 44, −36, 26; cluster size = 130; F(3,66) = 7.29, *FWE p* = 0.010, Fig. 1a). Post-hoc comparisons on the extracted beta values revealed that the HE group had significantly higher activation in the insular cortex relative to men (left: *p* = 0.002, right: *p* < 0.001) and OC users (left: *p* = 0.267, right: *p* = 0.020) (Fig. 1a lower panel). During late extinction, a main effect of Group was revealed in the anterior region of the left insular cortex (−24, 24, 2; cluster size = 70; F(3,62) = 7.88, *FWE p* = 0.004, Fig. 1b upper panel). Post-hoc analyses performed on the extracted beta values revealed that HE women had significantly greater activation in that region when compared to men (*p* = 0.007) and LE women (*p* = 0.024) (Fig. 1b lower panel). During the early extinction recall phase, a main effect of Group was revealed in the insular cortex bilaterally (left: −42, 4,10; cluster size  = 17; F(3,63) = 4.94, *FWE p* = 0.023, right: 50, −2, 8; cluster size = 6; F(3,63) = 3.066, *FWE p* = 0.013 (data for right not shown). Post-hoc analyses showed that HE women had significantly greater activation in that region when compared to men (left: *p* = 0 < 0.001, right: *p* = 0.001, Fig. 1c lower panel).

### Sex differences in the cingulate cortex

**Fig. 2:**
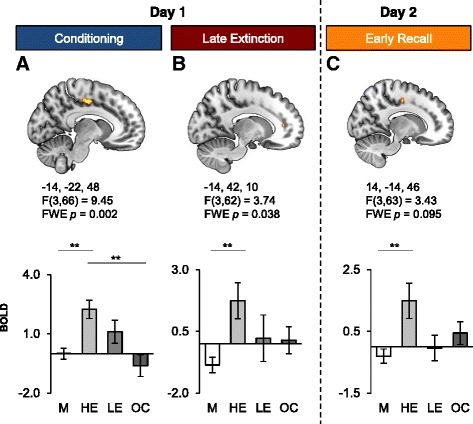
Cingulate cortex activations as a function of hormonal status grouping and phases of the fear conditioning and extinction paradigm. Significant functional activations of the cingulate cortex are displayed as a function of the different phases: fear conditioning (**a**, MCC), late extinction learning (**b**, rACC), and early extinction recall (**c**, MCC). The Montreal Neurological Institute (MNI) coordinates are listed below each functional activation, along with the corresponding statistics (note that the reported p values are FWE corrected). The lower panel indicates the BOLD signal extracted from the corresponding cluster as a function of the different groups during conditioning (**a**, MCC), late extinction (**b**, rACC), and early recall (**c**, MCC). FWE: family-wise error, M: men, HE: high estradiol women, LE: low estradiol women, OC: oral contraceptive users, MCC: middle cingulate cortex, rACC: rostral anterior cingulate cortex. Significant differences are noted (* *p* < 0.05 ** *p* < 0.01)

Significant group effects were observed in various sub-regions of the cingulate cortex (CC) as a function of the experimental phase (Fig. 2). During fear conditioning, a main effect of Group was revealed in the MCC (−14, −22, 48; cluster size = 200; F(3,66) = 9.45, *FWE p* = 0.002). Post-hoc analyses showed that the HE group had higher MCC activation during conditioning when compared to men (*p* < 0.001) and to OC users (*p* < 0.001) (Fig. 2a lower panel). During late extinction, the rostral portion of the cingulate cortex (rACC) showed a significant effect of Group (−14, 42, 10; cluster size = 6; F(3,62) = 3.74, *FWE p* = 0.038). Post-hoc analyses revealed that HE women had significantly higher activation in the rACC relative to men (*p* < 0.001, Fig. 2b lower panel). During early recall, there was a trend toward significance in the MCC (14, −14, 46; cluster size = 14; F(3,63) = 3.43, *FWE p* = 0.095), which was driven by HE women having significantly higher activation in that region relative to men (*p* = 0.001, Fig. 2c lower panel).

### Sex differences in other regions during fear acquisition

**Fig. 3:**
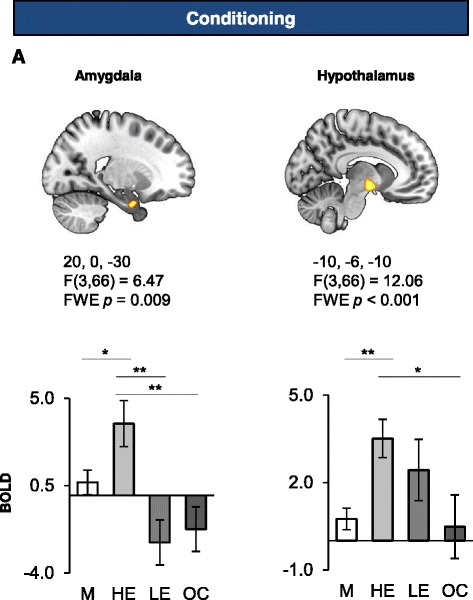
Amygdala and hypothalamus activations as a function of hormonal status grouping during fear conditioning. Significant functional activations of the amygdala (**a**) and the hypothalamus (**b**) during fear conditioning are displayed. The Montreal Neurological Institute (MNI) coordinates are listed below each functional activation, along with the corresponding statistics (note that the reported p values are FWE corrected). The lower panel indicates the BOLD signal extracted from the corresponding cluster as a function of the different groups during conditioning for the amygdala (**a**) and the hypothalamus (**b**). FWE: family-wise error, M: men, HE: high estradiol women, LE: low estradiol women, OC: oral contraceptive users. Significant differences are noted (*p < 0.05 **p < 0.01)

The amygdala and hypothalamus both showed significant group differences only during fear learning (amygdala: 22, 0, −30; cluster size = 50; F(3,66) = 6.47, *FWE p* = 0.009, Fig. 3a; hypothalamus: −10, −6, −10; cluster size = 181; F(3,66) = 12.06, *FWE p* < 0.001, Fig. 3b). Post-hoc analyses revealed that HE women exhibited significantly higher activation in the amygdala compared to all other groups (*p* = 0.018 for men, *p* = 0.001 for LE, and *p* = 0.003 for OC, Fig. 3a lower panel). With regards to the hypothalamus, post-hoc analyses revealed significantly higher activation in the HE group compared to both men (*p* < 0.001) and to OC users (*p* = 0.02) (Fig. 3b lower panel).

### Sex differences in response to shock delivery

**Fig. 4:**
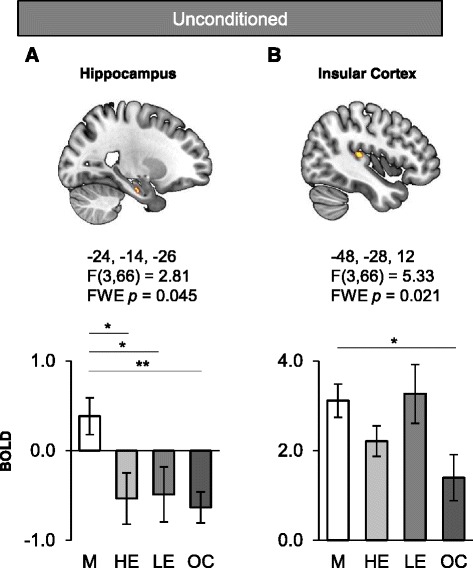
Hippocampus and insular cortex activations as a function of hormonal status grouping in response to shock delivery. Significant functional activations of the hippocampus (**a**) and the insular cortex (**b**) during shock delivery of the fear-conditioning phase are displayed. The Montreal Neurological Institute (MNI) coordinates are listed below each functional activation, along with the corresponding statistics (note that the reported p values are FWE corrected). The lower panel indicates the BOLD signal extracted from the corresponding cluster as a function of the different groups during shock delivery for the hippocampus (**a**) and the insular cortex (**b**). FWE: family-wise error, M: men, HE: high estradiol women, LE: low estradiol women, OC: oral contraceptive users. Significant differences are noted (*p < 0.05 **p < 0.01)

During the Shock vs. CS- offset contrast of the fear-conditioning phase, the hippocampus (left: −24, −14, −26; cluster size = 1; F(3,66) = 2.81, *FWE p* = 0.045, Fig. 4a) and the posterior insular cortex (−42, −28, 12; cluster size = 40; F(3,66) = 5.33, *FWE p* = 0.021, Fig. 4b) both yielded a main effect of Group. The post-hoc analyses indicated that men had significantly higher activation in the hippocampus relative to all other groups during the unconditioned response (*p* = 0.015 for HE, *p* = 0.03 for LE, and *p* = 0.007 for OC, Fig. 4a lower panel). Similarly, men also exhibited significantly higher activation in the posterior insular cortex relative to OC users during that same phase (*p* = 0.017, Fig. 4b lower panel).

### Brain activations associated with estradiol levels (Table 2)

**Table 2:** Brain regions activations that correlate with estradiol levels in naturally cycling women throughout the different experimental phases of the paradigm

Experimental phase	Correlation	Brain regions	x	y	z	Cluster size	t value	*p* value FWE
Fear conditioning	Positive	Posterior cingulate cortex	12	−34	46	70	3.10	0.033
		Posterior insular cortex	44	−38	24	116	3.22	0.034
		Amygdala	16	2	12	140	3.32	0.037
		Hypothalamus	−12	0	−8	33	2.71	0.039
		Anterior insular cortex	36	28	6	138	3.09	0.051
	Negative	Orbitofrontal cortex	−16	34	−24	21	3.21	0.038
		Subgenual anterior cingulate cortex	−6	18	−18	48	3.00	0.039
Shock delivery (unconditioned responses)	Positive	Hypothalamus	8	4	−32	357	4.11	0.015
		Middle insular cortex	−40	2	8	311	3.53	0.040
	Negative	Orbitofrontal cortex	−14	34	−18	44	3.40	0.011
Late extinction learning	Positive	Subgenual anterior cingulate cortex	−14	26	−14	30	2.88	0.028
	Negative	Middle insular cortex	−36	4	16	62	3.27	0.024
Early extinction recall	Positive	Parahippocampus	−30	2	−20	183	5.35	0.001
		Subgenual anterior cingulate cortex	10	12	−18	64	4.39	0.002
		Anterior insular cortex	32	18	12	82	4.35	0.003
		Anterior insular cortex	−62	10	0	1595	5.34	0.005
		Dorsal anterior cingulate cortex	10	24	40	91	4.13	0.005
		Ventromedial prefrontal cortex	−14	40	−26	572	4.37	0.014
		Middle cingulate cortex	−4	0	36	76	3.33	0.024
		Orbitofrontal cortex	−16	52	−18	572	3.94	0.034

For each experimental phase (fear conditioning, unconditioned responses ‘shock delivery’, late extinction learning and early extinction recall), correlations between brain activations and estradiol levels are listed. ‘Positive’ and ‘negative’ refer to the direction of the correlation. For each brain region, Montreal Neurological Institute (MNI) coordinates are reported along with cluster size, t value and p value (family-wise error corrected). Note that all these analyses were performed only in naturally cycling women (excluding men and women using oral contraceptives). Only brain regions from the fear conditioning and extinction networks are reported

During fear conditioning, a positive correlation was found between estradiol levels and the following brain regions: posterior cingulate cortex (12, −34, 46; cluster size = 70, t(24) = 3.10, *FWE p =* 0.033), posterior IC (44, −38, 24; cluster size = 116, t(24) = 3.22, *FWE p* = 0.034), amygdala (16, 2, −12; cluster size = 140, t(24) = 3.32, *FWE p =* 0.037), hypothalamus (−12, 0, −8; cluster size = 33, t(24) = 2.71, *FWE p =* 0.039) and anterior IC (36, 28, 6; cluster size = 138, t(24) = 3.09, *FWE p =* 0.051). A negative correlation was found during conditioning in the following regions: orbitofrontal cortex (−16, 34, −24; cluster size = 21, t(24) = 3.21, *FWE p =* 0.038) and subgenual ACC (−6, 18, −18; cluster size = 48, t(24) = 3.00, *FWE p =* 0.039).

For the unconditioned responses, a positive correlation was found between estradiol levels and the hypothalamus (8, 4, −32; cluster size = 357, t(24) = 4.11, *FWE p =* 0.015) as well as the middle IC (−40, 2, 8; cluster size = 311, t(24) = 3.53, *FWE p =* 0.040). There was, however, a negative correlation detected in the orbitofrontal cortex (−14, 34, −18; cluster size = 44, t(24) = 3.40, *FWE p =* 0.011).

During late extinction, a positive correlation was found between estradiol levels and the subgenual ACC (−14, 26, −14; cluster size = 30, t(22) = 2.88, *FWE p =* 0.028) whereas a negative correlation was found in the IC (−36, 4, 16; cluster size = 62, t(22) = 3.27, *FWE p =* 0.024).

During early extinction recall, positive correlations were found in the following brain regions: parahippocampus (−30, 2, −20; cluster size = 183, t(24) = 5.35, *FWE p =* 0.001), subgenual ACC (10, 12, −18; cluster size = 64, t(24) = 4.39, *FWE p =* 0.002), anterior IC (right: 32, 18, 12; cluster size = 82, t(24) = 4.35, *FWE p =* 0.003 and left: −62, 10, 0; cluster size = 1595, t(24) = 5.34, *FWE p =* 0.005), dorsal ACC (10, 24, 40; cluster size = 91, t(24) = 4.13, *FWE p =* 0.005), vmPFC (−14, 40, −26; cluster size = 572, t(24) = 4.37, *FWE p =* 0.014), MCC (−4, 0, 36; cluster size = 76, t(24) = 3.33, *FWE p =* 0.024), and orbitofrontal cortex (−16, 52, −18; cluster size = 572, t(24) = 3.94, *FWE p =* 0.034). No significant negative correlations were yielded between serum estradiol levels and brain activations during early extinction recall.

## Discussion

In this study, we evaluated sex differences as a function of hormonal status in the neural correlates of conditioned and unconditioned fear responses, extinction learning, and extinction recall. In fact, measuring estradiol levels in women enabled us to examine how variance in levels of this gonadal hormone may contribute to observed sex differences during fear conditioning and extinction. We observed significant effects of hormonal status across the different phases of the experiment and in different sub-regions of the insular and cingulate cortices, amygdala, hippocampus, and hypothalamus. Our data showed that the high estradiol (HE) group was the major contributor to these significant effects, with women from that group exhibiting higher activation in these brain regions during fear conditioning, late extinction learning, and early extinction recall. For most of the effects, HE women had significantly higher activation than men. In some instances, they also significantly differed from LE and OC women. During the shock delivery of the fear-conditioning phase, the pattern was somewhat different; men exhibited significantly higher activations (for a summary of the results, see Table 3). To complete these results, we have also performed regression analyses that allowed to pinpoint activations of brain regions that correlated with estradiol levels in naturally cycling women across the different phases. Taken together, these results confirm that the brain structures known to be key for fear learning and extinction are not only sexually dimorphic, but are also modulated by estradiol levels.

**Table 3:** Summary table for subgroup comparisons throughout the different experimental phases of the paradigm

	Unconditioned	Conditioning	Extinction	Recall
Insular cortex	M > OC	HE > M	HE > M	HE > M
		HE > OC	HE > LE	
MCC	n.s.	HE > M	n.s.	HE > M
		HE > OC		
rACC	n.s.	n.s.	HE > M	n.s.
Amygdala	n.s.	HE > M	n.s.	n.s.
		HE > LE		
		HE > OC		
Hypothalamus	n.s.	HE > M	n.s.	n.s.
		HE > OC		
Hippocampus	M > OC	n.s.	n.s.	n.s.

‘Unconditioned’ refers to the contrast Shock vs. CS- offset during fear conditioning, ‘Conditioning’ refers to the CS+ vs. CS- contrast during fear conditioning, ‘Extinction’ refers to the late CS+ vs. late CS- of the extinction training, and ‘Recall’ refers to the early CS + E trials vs. early CS + U trials of the extinction recall test. (n.s. = no significant main effect of group)

The elevated responses in different sub-regions of the insular and cingulate cortices in HE women are consistent with previous reports. For example, we have previously reported elevated vmPFC as well as dACC activations in HE women compared to LE women [24]. Elevated activation in the arousal circuitry, which includes the hypothalamus, the amygdala, and the cingulate cortex, has also been documented in HE women in response to emotional stimuli [7]. The elevated activation reported in this study in HE women may be related to enhanced memory consolidation associated with the different phases of the experiment. High levels of estradiol have been shown to enhance memory consolidation in a number of different paradigms as well as enhance long-term potentiation in hippocampal neurons during contextual fear learning [17, 24].

We have previously shown that, at the psychophysiological level, the magnitude of extinction memory was comparable between men and HE women, and that both of these groups showed superior extinction retention relative to LE women [29]. We have also shown that extinction memory was significantly reduced in women using oral contraceptives compared to HE women [30]. Based on these data, we had anticipated that brain activations between men and the HE group would be the most comparable whereas the LE and OC groups would differ the most from men. The findings, however, were not consistent with our predictions nor with our previously reported psychophysiological data from the same women analyzed in the current study. One possible explanation for this finding is that men and women achieve the same behavioral outcome but by using different neurobiological networks. These important data highlight the need of understanding how men and women consolidate fear-related memories differently so that treatment for anxiety and mood disorders may be specifically tailored to the different sexes, while taking into account the hormonal status.

LE and OC women exhibit similar brain activations, yet their estradiol levels differ: LE women have temporarily low levels of natural estradiol whereas OC women have both constantly low levels of natural estradiol and constantly elevated levels of synthetic estradiol. These data suggest that the cyclicity of estradiol may be key in enhancing the activation of brain regions involved in both fear learning and extinction. An alternative interpretation is that endogenous estradiol may have a different or more efficacious influence on this brain network activation when compared to synthetic estradiol. A growing amount of research demonstrates that OC use may lead to structural modifications in brain regions implicated in high-order cognitive function [35], including regional gray matter volumes in regions associated with the fear extinction network such as the prefrontal cortex, anterior cingulate gyrus, cerebellum, and the hippocampus [36, 37]. Several studies have noted functional differences in OC users during the resting state [38] as well as during face and reward processing [39, 40]. A recent study reported that OC users performed behaviorally like naturally cycling women during two numerical tasks, but actually displayed male-like brain-activation patterns [41]. This apparent discrepancy is similar to our findings showing a discrepancy between the OC users’ behavior and their brain activation patterns, in which there may be a somewhat ‘masculinizing’ effect of OC use on brain activation. Together, these data suggest that OC use may cause structural reorganization and lead to differences in activation in response to a wide array of tasks; and this may in turn induce changes in behavioral output and brain activation. Beyond such research implications, this area of study is also critical because there appear to be effects of OC use on brain structure, function, and behavioral output. Notably, 80 % of women of reproductive age in the United States use oral contraceptives for birth control, with the average age of first OC use rapidly decreasing into the early years of adolescence, a time especially sensitive to neuroplasticity [42].

We note a few limitations of the present study that should be considered. First, we did not collect other hormonal data, such as cortisol levels. In fact, cortisol has been shown to interact with estrogens and influence sex differences, fear learning, and fear extinction [43]. Second, the effects reported in this manuscript pertain to monophasic OC users. These factors should therefore be considered in future work attempting to replicate and build upon the findings of the present study.

Despite these limitations, a key contribution of the present study is to highlight the potential to entirely overlook sex differences in the neurobiology of fear extinction based upon the composition of the sample of women tested. Our findings of multiple HE-driven sex differences would have been occluded if our sample population consisted of mainly OC users and/or LE women. This finding may also contribute to discrepancies between studies in this area of research in which some reports note sex differences and others do not. Our paper emphasizes the importance of considering whether or not women are naturally cycling, what their hormone levels are during their participation, and their use of hormonal contraceptives when examining the neurobiology of fear extinction.

## Conclusions

The present study demonstrates that estradiol levels may influence the degree of activation in the brain’s fear-extinction network. It is therefore important to consider gonadal hormonal status, oral contraceptive use, and menstrual cycle phase when investigating sex differences in the context of fear conditioning, fear extinction, and other emotional regulation tasks. As these factors contribute to the endocrine milieu, taking them into account may serve to reduce the variability and seemingly contradictory findings among different studies on sex differences in emotion regulation.

## Abbreviations

ACC: Anterior cingulate cortex
BOLD: Blood oxygenation level dependent
CC: Cingulate cortex
CS: Conditioned stimulus
dACC: Dorsal anterior cingulate cortex
fMRI: Functional magnetic resonance imaging
FWE: Family-wise error
HE: High estradiol
IC: Insular cortex
LE: Low estradiol
MCC: Middle cingulate cortex
MNI: Montreal Neurological Institute
OC: Oral contraceptives
rACC: Rostral anterior cingulate cortex
ROI: Region of interest
US: Unconditioned stimulus
vmPFC: Ventromedial prefrontal cortex
